# Prostate Cancer among Patients Undergoing Radical Cystoprostatectomy for Bladder Cancer in the Department of Urology in a Tertiary Care Centre

**DOI:** 10.31729/jnma.8309

**Published:** 2023-10-31

**Authors:** Prashant Mishra, Li Jiongming, Liu Jianhe, Fang Kewei, Jiang Yongming, Guang Wang, Li Pei, Yang Tongxing

**Affiliations:** 1 Department of Urology, The Second Affiliated Hospital of Kunming Medical University, Yunnan Institute of Urology, Kunming, Yunnan, People's Republic of China

**Keywords:** *bladder cancer*, *incidental findings*, *prostate cancer*

## Abstract

**Introduction:**

Prostate cancer is the most common malignancy in men and remains one of the most prevalent and least understood of all human malignancies. Bladder cancer is the most frequently diagnosed cancer in China. Radical cystectomy remains the gold standard for muscle-invasive, recurrent and multiple bladder cancer. All male patients undergoing radical cystoprostatectomy must be evaluated for prostate cancer before planning surgery. The aim of this study was to find out the prevalence of prostate cancer among patients undergoing radical cystoprostatectomy undergoing surgery for bladder cancer.

**Methods:**

A descriptive cross-sectional study was conducted in a tertiary care centre from 1 August 2023 to 30 August 2023 where data from 1 January 2015 to 30 December 2017 was taken from medical records after obtaining ethical approval from the Ethical Review Board. All patients who underwent radical cystoprostatectomy were included in the study. Whole radical cystoprostatectomy specimens were cut transversely at 3 mm intervals and examined in the same pathological centre. Clinically significant prostate cancer was defined as a tumour with a Gleason pattern ≥4, prostate tumour with clinical stage ≥pT3, lymph node. involvement, positive surgical margin or multifocality of three or more lesions. A convenience sampling method was used. The point estimate was calculated at a 95% Confidence Interval.

**Results:**

Among 210 patients, 52 (24.76%) (18.92-30.60, 95% Confidence Interval) had incidental prostate cancer. The average age of patients with incidental prostate cancer was 65.88±9.54 years.

**Conclusions:**

The prevalence of incidental prostate cancer was found to be lower than the study conducted in a similar setting.

## INTRODUCTION

Prostate cancer (PCa) is the most common malignancy in men.^1^ Autopsy studies show up to 30% of men in 50 years have incidental prostate cancer (IPCa) and increases to 70% in 70-80 years, rare below 45 years. Bladder cancer (BCa) is the second most common genitourinary malignancy and frequently diagnosed cancer in China. Radical cystectomy remains the gold standard for bladder cancer.^2^

Involvement of adenocarcinoma in the prostate has been commonly observed during pathological examination of radical cystoprostatectomy (RCP) specimens. IPCa in RCP specimens is very common in Europe and the USA. The highest rate reported was 60%.^3^ Rate is still lower in the Asian population, with prevalence of 28%, 18.1% and 36.5%.^4^ All male patients undergoing radical cystoprostatectomy must be evaluated for prostate cancer before planning for surgery.

The aim of this study was to find out the prevalence of prostate cancer among patients undergoing radical cystoprostatectomy undergoing surgery for bladder cancer.

## METHODS

A descriptive cross-sectional study was conducted at the Department of Urology, The Second Affiliated Hospital of Kunming Medical University, Yunnan Institute of Urology, Kunming, Yunnan, People's Republic of China from 1 August 2023 to 30 August 2023 to where data from 1 January 2015 to 30 December 2017 was taken from medical records after obtaining ethical approval from the Ethical Review Board (Reference number: 2023-162). Patients with bladder cancer undergoing RCP for muscle-invasive recurrent and multiple transitional cell carcinoma of bladder were taken. Convenience sampling method was used. The sample size was calculated using the following formula:


$$\begin{array}{ll} \text{n} & ={\text{Z}}^{2}\times \frac{\text{p}\times \text{q}}{{\text{e}}^{\text{2}}}\\ & =1.96^{2}\times \frac{0.28\times 0.72}{0.07^{2}}\\ & =158 \end{array}$$

Where,

n = minimum required sample size
Z = 1.96 at a 95% Confidence Interval (CI)
p = prevalence of incidental prostate cancer taken from previous study, 28%^4^
q = 1-p
e = margin of error, 7%

Thus, the calculated minimum required sample size was 158. However, 210 patients were taken for the study.

All patients from the Department of Urology undergoing RCP for BCa were taken. None of the patients had a known history of prostate cancer before surgery or a history of radiotherapy or chemotherapy. Preoperative evaluation was done, which included prostate specific antigen level (PSA), digital rectal examination (DRE), chest X-ray, B ultrasound, intravenous pyelogram and either computed tomography (CT) scan or magnetic resonance imaging (MRI) scan. The patient's medical history and histopathological examination were reviewed in order to identify men with incidental carcinoma of the prostate.

The radical cystoprostatectomy specimens were routinely handled according to the standard procedures. The RCP specimens, which consist of bladder, prostate, and seminal vesicles when they were received in the pathology laboratory were put into adequate amount of 10% neutral formalin for fixation for 24 hours. The prostate and seminal vesicles were removed en bloc from the bladder. Each of the specimens was then examined by the pathologist and the tissue blocks were sampled separately. These tissue blocks were processed, embedded in paraffin and sections were cut transversely at a thickness of 3 mm intervals. The sections were put on slides and were stained with hematoxylin and eosin. Tissue samples of each crosssection were examined under a microscope. The TNM classification was used for the pathological staging and the World Health Organisation (WHO) classification for histopathological typing and grading.^5^

Clinically significant prostate cancer was defined according to standard criteria,^6^ which states that incidental prostate cancer will be considered significant if any of the following are present: total tumor volume 0.5 cc or more; Gleason grade 4 or more; extraprostatic extension; seminal vesicle invasion; lymph node metastasis (of prostate cancer); or positive surgical margin.

Data was entered and analysed using IBM SPSS Statistics version 23.0. The point estimate was calculated at a 95% CI.

## RESULTS

Among 210 patients, 52 (24.76%) (18.92-30.60, 95% CI) had incidentally diagnosed prostate cancer. The average age of patients with incidental prostate cancer was 65.88±9.54 years. A tumor volume of more than 5% was identified in 11 (21.15%) patients. The Gleason score of 7 was seen in 8 (15.38%) patients, Gleason score 8 in 1 (1.92%) patient, Gleason 6 in 40 (76.92%) and Gleason 4 and 5 in 3 (5.77%). High-grade prostatic intraepithelial neoplasia (HGPIN) was seen positive in 26 (50%) patients. Multifocality is seen in 2 (3.85%) patients (Table 1).

**Table 1:** Details of patients with incidental prostate cancer (n= 52).

Parameters	Total incidental PCa n (%)
**TNM stage PCa**	
pT2a	46 (88.46)
pT2b	3 (5.77)
pT3a	3 (5.77)
**Gleason score**	
2+2	1 (1.92)
3+2	2 (3.85)
3+3	40 (76.92)
3+4	5 (9.61)
4+3	3 (5.77)
4+4	1 (1.92)
**Tumor volume**	
<5%	38 (73.08)
>5%	11 (21.15)
**Surgical margin**	
Positive	5 (9.61)
Negative	47 (90.38)
**Bladder cancer TNM staging**	
TIS	1 (1.92)
pT1a	12 (23.08)
pT2a	15 (28.85)
pT2b	12 (23.08)
pT3a	11 (21.15)
pT3b	1 (1.92)

A total of 12 (23.07%) patients were diagnosed as clinically significant prostate cancer (Table 2).

**Table 2:** Details of patients with significant prostate cancer (n= 12).

Parameters	n (%)
**TNM stage PCa**	
pT2a	7 (58.33)
pT2b	2 (16.67)
pT3a	3 (25)
**Gleason score**	
2+2	-
3+2	-
3+3	3 (25)
3+4	5 (41.67)
4+3	3 (25)
4+4	1 (8.33)
**Tumor volume**	
<5%	6 (50)
>5%	6 (50)
**Surgical margin**	
Positive	5 (41.67)
Negative	7 (58.33)
**Bladder cancer TNM staging**	
TIS	-
pT1a	5 (41.67)
pT2a	1 (8.33)
pT2b	3 (25)
pT3a	3 (25)
pT3b	-

A total of 40 (76.92%) patients had clinically insignificant prostate cancer (Table 3).

**Table 3:** Details of patients with insignificant prostate cancer (n= 40).

Parameters	n (%)
**TNM stage PCa**	
pT2a	39 (97.50)
pT2b	1 (2.50)
pT3a	-
**Gleason score**	
2+2	1 (2.50)
3+2	2 (5)
3+3	37 (92.50)
3+4	-
4+3	-
4+4	-
**Tumor volume**	
<5%	32 (80)
>5%	5 (12.50)
**Surgical margin**	
Negative	40 (100)
**Bladder cancer TNM staging**	
TIS	1 (2.50)
pT1a	7 (17.50)
pT2a	14 (35)
pT2b	9 (22.50)
pT3a	8 (20)
pT3b	1 (2.50)

## DISCUSSION

Among 210 patients, 52 (24.76%) had incidentally diagnosed prostate cancer which is similar to a study^4^ but this is significantly lower than that of western population. In a study conducted at London shown that the prevalence of PCa was 60%^3^, similarly another study was done in Italy showed that the prevalence was 49.6%.^7^

As for the pathological features of the incidental prostate cancer, our study showed that most of them was pT2a at 88.46%, with a Gleason score 6 in 76.92% suggesting a more favourable prognosis, which was similar to the study conducted in the United States, Europe, and Asia.^8-10^ In a study done in 2014, incidental finding of 23% was seen which is also similar to our study.^11^ Our retrospective study, as with other studies cited above, is exposed to selection bias. PSA testing was limited in the pre-operative period probably due to thinking of that muscle-invasive bladder Ca is a more serious disease, and the majority of incidental PCa would be clinically insignificant. The frequency of incidental prostate cancer done in China was 3.3%,^12^ somewhat similar to the frequency in Taiwan^13^ and in Japan^14^ (4% and 5.1% respectively). Similarly, a study done in China reported the incidence of 7%, which is similar to the incidence in India.^15,16^

In China, frequency of incidental prostate cancer is low as reported by several authors but some other have also reported higher frequency,^4^ which reported 28% of incidental prostate cancer. Even other Asia countries have similar frequency as China except report from Korea^17^ reported 36.5%, which is much higher than China and other Asian countries. Such an obvious difference of prostate cancer prevalence between China or some other Asian countries and Western countries might be relating to genetic backgrounds, lifestyles, socio-economic status and diet which is a contributing factor in aggravating genomic susceptibility of prostate cancer. The various geographical distribution across the world implies that the genetic background of prostate cancer might play a role in causing cancer. In addition, males with history of prostate cancer in relatives of the first and second generation are highly susceptible to get prostate cancer. The studies of twins demonstrated that monozygotic twins have a significantly higher risk than dizygotic in twins. Even though African Americans are highly susceptible to prostate cancer in United States but in proper African nationals have remarkably lower incidence,^18^ which also suggests that other important factors may also contribute and influence the incidence of prostate cancer occurrence. Meanwhile, the significant difference of the lifestyle and dietary habits might also relate to prostate cancer incidence. Recent study revealed that the risk of prostate cancer was inversely associated with total dietary fiber intake, insoluble and legume fiber intakes, which are the staple diet in many Asian countries.^19^ Whereas, green tea might also contribute in reducing prostate cancer risk, which is a most popular drink in China and Japan. A study of green tea and cancer prevention showed a decreased risk of prostate cancer in men consuming higher quantities green tea or green tea extracts.^20^

This study had some limitations. This was a single centre study due to which the study could not be generalised.

## CONCLUSIONS

The prevalence of incidental prostate cancer was found to be lower than the study conducted in a similar setting. Long term follow-up of patients with incidentally detected PCa especially those with significant incidentally PCa is required to assess if they develop biochemical or clinical progression of disease.
